# Integrating Metabolomics and Machine Learning to Analyze Chemical Markers and Ecological Regulatory Mechanisms of Geographical Differentiation in *Thesium chinense* Turcz

**DOI:** 10.3390/metabo15070423

**Published:** 2025-06-20

**Authors:** Cong Wang, Ke Che, Guanglei Zhang, Hao Yu, Junsong Wang

**Affiliations:** 1 Center for Molecular Metabolism, School of Environmental and Biological Engineering, Nanjing University of Science and Technology, 200 Xiao Ling Wei Street, Nanjing 210094, China; 2 College of Food Science and Engineering, Anhui Science and Technology University, Chuzhou 233100, China; cheke1998818@163.com (K.C.); 13339228318@163.com (G.Z.); 3 School of Chinese Medicine, Bozhou University, Bozhou 236800, China

**Keywords:** *Thesium chinense* Turcz., metabolomics, geo-authentic herbs, machine learning, antioxidant activity, environmental factors, UHPLC-Q-TOF/MS

## Abstract

Background: The relationship between medicinal efficacy and the geographical environment in *Thesium chinense* Turcz. (*T. chinense* Turcz.), a traditional Chinese herb, remains systematically unexplored. This study integrates metabolomics, machine learning, and ecological factor analysis to elucidate the geographical variation patterns and regulatory mechanisms of secondary metabolites in *T. chinense* Turcz. from Anhui, Henan, and Shanxi Provinces. Methods: Metabolomic profiling was conducted on *T. chinense* Turcz. samples collected from three geographical origins across Anhui, Henan, and Shanxi Provinces. Machine learning algorithms (Random Forest, LASSO regression) identified region-specific biomarkers through intersection analysis. Metabolic pathway enrichment employed MetaboAnalyst 5.0 with target prediction. Antioxidant activity (DPPH/hydroxyl radical scavenging) was quantified spectrophotometrically. Environmental correlation analysis incorporated 19 WorldClim variables using redundancy analysis, Mantel tests, and Pearson correlations. Results: We identified 43 geographical marker compounds (primarily flavonoids and alkaloids). Random forest and LASSO regression algorithms determined core markers for each production area: Anhui (4 markers), Henan (6 markers), and Shanxi (3 markers). Metabolic pathway enrichment analysis revealed these markers exert pharmacological effects through neuroactive ligand–receptor interaction and PI3K-Akt signaling pathways. Redundancy analysis demonstrated Anhui samples exhibited significantly higher antioxidant activity (DPPH and hydroxyl radical scavenging rates) than other regions, strongly correlating with stable low-temperature environments (annual mean temperature) and precipitation patterns. Conclusions: This study established the first geo-specific molecular marker system for *T. chinense* Turcz., demonstrating that the geographical environment critically influences metabolic profiles and bioactivity. Findings provide a scientific basis for quality control standards of geo-authentic herbs and offer insights into plant–environment interactions for sustainable cultivation practices.

## 1. Introduction

*Thesium chinense* Turcz. (*T. chinense* Turcz.), long valued for centuries in traditional Chinese medicine (TCM) for its heat-clearing, detoxifying, kidney-nourishing, and essence-securing properties, is documented in classical texts like the *Compendium of Materia Medica* for its use in treating inflammation, infections, and skin conditions [1,2]. Modern formulations (e.g., Bairui tablets) leverage its bioactive compounds—including flavonoids (potent antioxidants), alkaloids (broad antimicrobials), coumarins, and polysaccharides—to combat respiratory infections and pharyngitis. These compounds underpin its anti-inflammatory, antimicrobial, antioxidant, and immunomodulatory activities, working through mechanisms like oxidative stress alleviation, ferroptosis inhibition, and inflammatory factor modulation [3,4].

Modern metabolomics platforms, particularly ultra-performance liquid chromatography-quadrupole time-of-flight mass spectrometry (UHPLC-Q-TOF/MS), have revolutionized the study of medicinal plants like *T. chinense* Turcz., enabling the high-throughput detection of hundreds of metabolites and providing unprecedented insights into their chemical diversity [5]. These advancements have also started to transform empirical concepts like “geo-authenticity” into scientifically assessable frameworks, grounded in molecular markers, component networks, pathway regulation, and mechanisms of environmental adaptation [6]. Geo-authenticity is the principle that herbs cultivated in specific geographical regions exhibit superior quality and efficacy due to the influence of unique environmental conditions.

Despite the growing body of research on the chemical constituents of *T. chinense* Turcz., significant knowledge gaps remain regarding the geographical variation of this species and its direct impact on chemical composition, bioactivity, and, ultimately, therapeutic efficacy and quality.

Current studies predominantly focus on established geo-authentic regions such as Anhui and Henan, often employing limited analytical strategies, such as single-component quantification (e.g., HPLC for rutin), without comprehensive multi-regional comparisons [7]. While some studies suggest that Anhui-sourced material may contain higher levels of saccharides, saponins, and flavonoids due to local climate and soil conditions [8], a systematic understanding of the mechanisms underlying geo-authenticity and the stability of key bioactive components across major production regions is lacking. This gap fundamentally hinders robust quality control, reliable clinical application, and effective market authentication.

Moreover, conventional chemometric methods like principal component analysis (PCA) are often inadequate for deciphering the complex, nonlinear relationships inherent in modern metabolomic datasets. These methods frequently fail to accurately identify region-specific core biomarkers or to quantify the intricate relationships between environmental factors, metabolites, and bioactivity that are crucial for understanding geo-authenticity [9]. Compounding this issue is the notable absence of systematic studies validating how specific climatic factors—such as temperature variability and precipitation patterns—along with altitude and soil properties, influence the biosynthesis of pharmacologically vital compounds, including alkaloids and flavonoids [10], across diverse geographical populations of *T. chinense* Turcz.

To address these critical gaps, this study employs an integrated approach that combines advanced metabolomics, climate response analysis, and bioactivity testing specifically targeting *T. chinense* Turcz. from three major production areas recognized for their geo-authenticity: Anhui, Henan, and Shanxi. Samples were systematically collected from multiple localities within each province, accompanied by detailed environmental data. UPLC-QTOF-MS analysis was utilized to generate comprehensive chemical profiles and identify region-discriminating metabolites. Sophisticated machine learning algorithms, including Random Forest (RF) and Least Absolute Shrinkage and Selection Operator (LASSO), were applied to screen for robust, region-specific core biomarkers from the complex metabolomic data. Additionally, the antioxidant capacity (DPPH and hydroxyl radical scavenging activity) of samples from each region was quantitatively assessed. Redundancy analysis (RDA) and Pearson correlation were then employed to elucidate the quantitative relationships between the identified environmental variables (e.g., mean annual temperature, precipitation seasonality), the relative abundance of key metabolites, and the measured antioxidant activities.

This research represents a pioneering systematic effort to establish a traceable, geo-specific molecular marker system for *T. chinense* Turcz. across its primary production regions. By directly linking environmental factors to specific chemical profiles and bioactivities, this study aims to fill the theoretical and data gaps concerning geographical adaptation and molecular tracing. The findings are expected to significantly enhance our scientific understanding of Chinese geo-authentic herbs, ultimately paving the way for improved quality control protocols, reliable standardization practices, and informed conservation strategies for *T. chinense* Turcz.

## 2. Materials and Methods

### 2.1. Sample Collection

**Figure 1:**
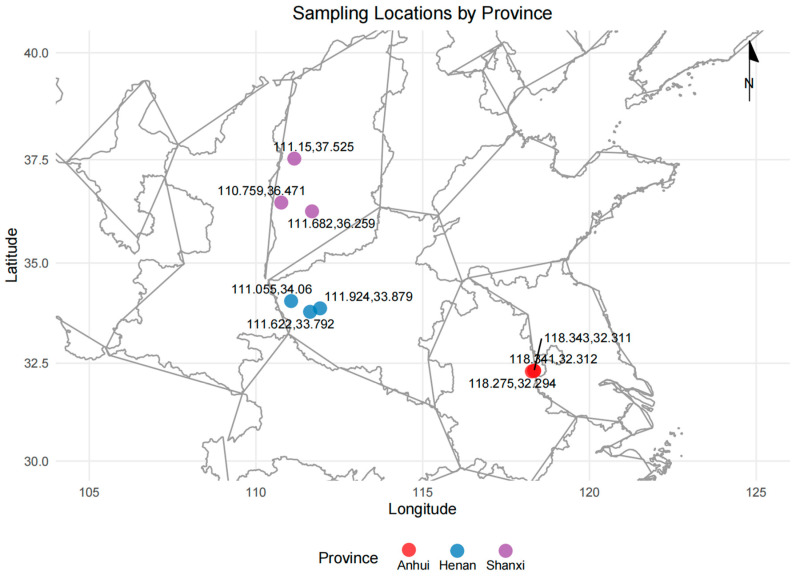
Detailed sample information of the geographical origins.

*T. chinense* Turcz. samples were collected from nine localities across three provinces in China (Anhui, Henan, and Shanxi) during September and October 2022 (Figure 1). Three samples were collected from each locality, selecting two-year-old plants with a length of 25–30 cm. The samples were identified by Professor Liu Hanzhen from Anhui University of Science and Technology and stored under the accession numbers 2022071901 (Anhui), 2022071902 (Henan), and 2022071903 (Shanxi) at the Center for Molecular Metabolism, Nanjing University of Science and Technology.

### 2.2. Metabolite Extraction

Fresh plant tissues were ground into fine powder in a liquid nitrogen-cooled mortar [5]. Approximately 15 mg of the tissue powder was accurately weighed and transferred into a 2 mL EP tube. A pre-cooled chloroform/water/methanol (20:20:60, *v*/*v*/*v*) mixture and tungsten carbide beads were added, and the mixture was homogenized at 70 Hz for three intermittent cycles (60 s each with a 5 s interval) using a low-temperature homogenizer. The homogenate was centrifuged at 12,000 rpm for 10 min at 4 °C. The supernatant was collected, dried under nitrogen, and re-dissolved in 500 μL of 50% methanol solution. The re-dissolved sample was vortexed for 30 s, followed by sonication in an ice bath for 1 min and centrifugation at 12,000 rpm for 15 min at 4 °C. The clear supernatant was transferred to a vial for UHPLC-MS injection. Quality control (QC) samples were also prepared to ensure the stability and reproducibility of the mass spectrometry.

### 2.3. LC-MS Detection Method

The LC-MS analysis was performed on a SCIEX Exion LC UHPLC-MS system coupled with a Triple TOF™ 5600+ mass spectrometer equipped with a DuoSpray ion source. Chromatographic separation was achieved using a Phenomenex Kinetex^®^ C18 column (100 × 2.1 mm, 2.6 μm) with a Security Guard UHPLC C18 guard column at 40 °C. The mobile phase consisted of 0.1% formic acid in water (*v*/*v*) and acetonitrile at a flow rate of 0.3 mL/min. Gradient elution was programmed as follows: 1% acetonitrile at 0–1 min, linear increase to 100% acetonitrile at 10 min, hold at 100% acetonitrile until 13 min, return to 1% acetonitrile at 14 min, and re-equilibration until 17 min. The autosampler temperature was maintained at 4 °C with an injection volume of 2 μL.

Mass spectrometric detection was carried out in both positive and negative ion modes with the following parameters: ion source temperature 550 °C, nebulizer gas (GS1) 55 psi, auxiliary gas (GS2) 55 psi, curtain gas 35 psi, ion spray voltage 5500 V (+mode)/−4500 V (−mode). For TOF MS-IDA-MS/MS acquisition, the mass range was set to 100–1250 *m*/*z* for full-scan MS (accumulation time 0.10 s) and 50–1250 *m*/*z* for product ion scans (accumulation time 0.05 s). The information-dependent acquisition parameters included the following: declustering potential 80 V (+)/−80 V (−), collision energy 35 ± 15 V (+)/−35 ± 15 V (−), ion intensity threshold 100 cps, isotope exclusion window 4 Da, mass tolerance 50 mDa, and 10 candidate ions monitored per cycle. Dynamic background subtraction was enabled to enhance trace analyte sensitivity. The system was automatically calibrated every 5 samples using the external calibration delivery system.

Data processing involved converting the raw files to mzML format using ProteoWizard software, followed by ion feature extraction using XCMS software. Metabolite identities were assigned by matching the spectra with the Human Metabolome Database (HMDB). Data normalization was performed using R, and further analysis was carried out using the mixOmics R package (version 6.30.0) [11,12]. Potential biomarkers were identified based on VIP scores and *t*-tests.

### 2.4. Machine Learning-Based Biomarker Screening

Random Forest (RF) [13] and Lasso regression [14] algorithms were employed to select region-specific biomarkers from the chemical compositions of the three regions. Intersection analysis was conducted to identify region-specific biomarkers, and statistical analysis was used to verify the stability of the screening results.

### 2.5. Pathway Analysis of Metabolites and Related Genes and Proteins

Metabolite set enrichment analysis was performed using MetaboAnalyst 5.0 (https://www.metaboanalyst.ca/, accessed on 16 May 2024) [15]. The KEGG database (http://www.kegg.jp/, accessed on 16 May 2024) [16] was used for pathway mapping. The components of *T. chinense* Turcz. were subjected to batch prediction of targets using TargetNet (http://targetnet.scbdd.com/, accessed on 16 May 2024), followed by GO and KEGG enrichment analysis.

### 2.6. Antioxidant Activity Assays

#### 2.6.1. DPPH Radical Scavenging Activity Assay

*T. chinense* Turcz. sample powder (50 mg) was mixed with DPPH solution and nitrogen radical extraction solution, and the absorbance was measured at 517 nm [17]. A control sample without DPPH solution was used for comparison. The DPPH scavenging rate was calculated.

#### 2.6.2. Hydroxyl Radical Scavenging Activity Assay

*T. chinense* Turcz. sample powder (100 mg) was extracted with 4 mL of methanol by ultrasonication. The supernatant was diluted and mixed with double-distilled water and substrate application solution, followed by incubation at 37 °C for 1 min. The reaction was terminated by adding a chromogenic agent [18], and the hydroxyl radical scavenging ability of the sample was determined.

### 2.7. Correlation Between T. chinense Turcz. Metabolites and Environmental Factors

A total of 19 environmental variables (11 temperature variables and 8 precipitation variables) were recorded for each sample area from the WorldClim Version 2 dataset (http://www.worldclim.org/, accessed on 16 May 2024) [19,20], as shown in Table 1.

Redundancy analysis (RDA) and Mantel tests were performed in R to explore the relationships between environmental factors, antioxidant activity, and *T. chinense* Turcz. metabolites. Pearson correlation analysis was used to associate the relative metabolite content with environmental factors and antioxidant activity.

## 3. Results

### 3.1. Differences in Chemical Composition and Metabolic Pathway Characteristics of Thesium chinense from Different Regions

Liquid chromatography-mass spectrometry (LC-MS) was used to systematically analyze the chemical composition of *T. chinense* Turcz. samples collected from Anhui, Henan, and Shanxi. A chemical fingerprint database was constructed, and differential biomarkers and secondary metabolic pathway characteristics were identified through multivariate statistics (PCA, PLS-DA, Heatmap) and metabolite set enrichment analysis.

**Figure 2:**
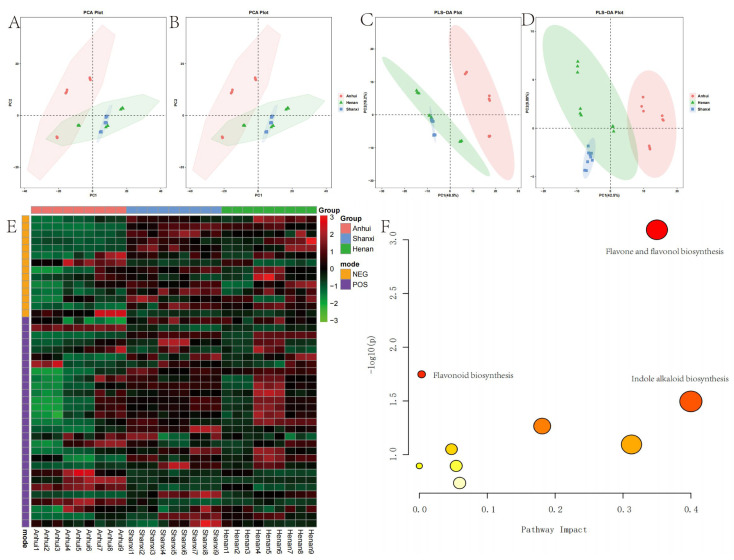
Multivariate analysis and metabolic pathway characteristics of *T. chinense* Turcz. samples from Anhui, Henan, and Shanxi. PCA score plots in positive (POS) (**A**) and negative (NEG) (**B**) ion modes; PLS-DA score plots in POS (**C**) and NEG (**D**) modes; (**E**) heatmap of differential metabolites; (**F**) metabolic pathway enrichment analysis.

The principal component analysis (PCA) model (Figure 2A,B) showed spatial overlap between Henan samples and the other two regions, with an overlap area of 63.5% with Shanxi samples, indicating high similarity in metabolite composition between these two regions. In contrast, the partial least squares discriminant analysis (PLS-DA) model (Figure 2C,D) exhibited stronger inter-group discrimination in positive ion mode, with Anhui samples forming a distinct cluster on the PC1 axis, suggesting significant specificity in metabolite composition for this region.

The heatmap analysis (Figure 2E) visually presented the relative abundance gradient distribution of 289 compounds in positive ion mode and 217 compounds in negative ion mode. Screening criteria of VIP ≥ 1 and *p* < 0.05 identified a total of 43 statistically significant differential metabolites. Metabolite set enrichment analysis revealed ten metabolic pathways (Figure 2F), with the top three being flavone and flavonol biosynthesis, flavonoid biosynthesis, and indole alkaloid biosynthesis. These pathways indicate significant metabolic activity in the synthesis of flavonoids and indole alkaloids in *T. chinense* Turcz. samples, which are closely related to their known pharmacological effects.

### 3.2. Geographical Impact on the Chemical Composition of T. chinense Turcz.

**Figure 3:**
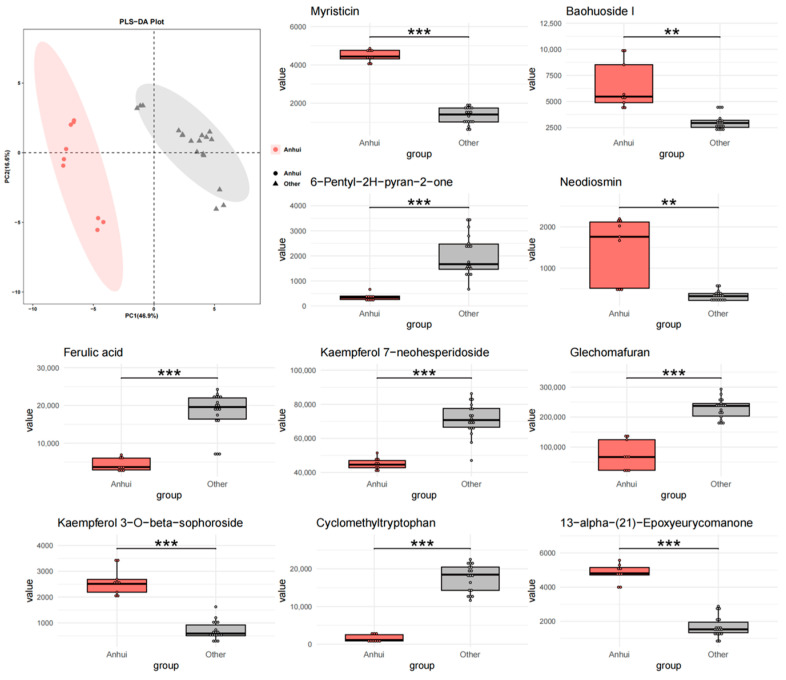
PLS-DA analysis and top 10 characteristic compounds of *T. chinense* Turcz. from Anhui vs. other regions. ** *p* < 0.01, *** *p* < 0.001, vs. Other group.

**Figure 4:**
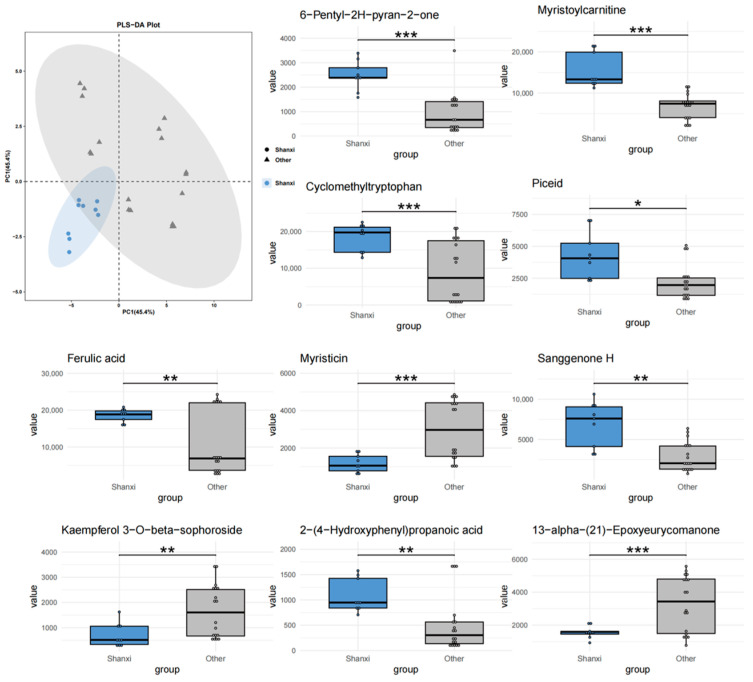
PLS-DA analysis and top 10 characteristic compounds of *T. chinense* Turcz. from Shanxi vs. other regions. * *p* < 0.05, ** *p* < 0.01, *** *p* < 0.001, vs. Other group.

**Figure 5:**
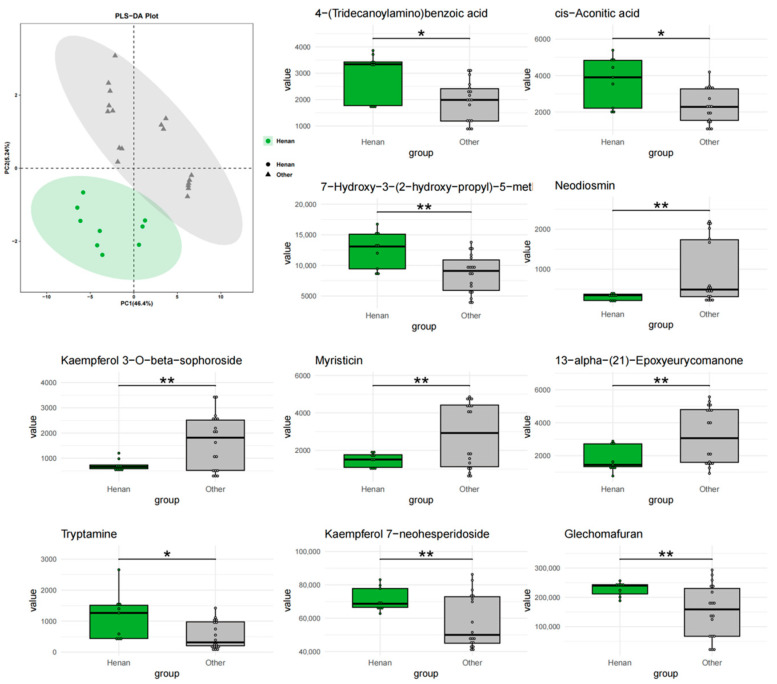
PLS-DA analysis and top 10 characteristic compounds of *T. chinense* Turcz. from Henan vs. other regions. * *p* < 0.05, ** *p* < 0.01, vs. Other group.

To further investigate the impact of geographical factors on the chemical composition of *T. chinense* Turcz., three comparative models were constructed (Anhui vs. others, Shanxi vs. others, Henan vs. others). PLS-DA analysis showed that Anhui samples (Figure 3) had no overlap with other regions, while Shanxi (Figure 4) and Henan (Figure 5) samples exhibited significant overlap with other regions, indicating that Anhui samples had the most unique chemical characteristics, whereas Shanxi and Henan samples had lower distinguishability from the other two regions.

At the level of differential metabolites, in the “Anhui vs. others” group, Glechomafuran and Cyclomethyltryptophan were significantly downregulated (*p* < 0.001), while Myristicin showed specific enrichment, but the expression was reversed in the Shanxi group. This cross-group expression heterogeneity may reflect the differential regulation of secondary metabolite synthesis by geographical environments. Kaempferol flavonoid metabolites exhibited bidirectional regulation, with Kaempferol 7-neohesperidoside abundance decreased by 82%, while its isomer Kaempferol 3-O-beta-sophoroside increased by 3.2-fold. This reverse regulation of homologous metabolites may be due to tissue-specific modifications in homologous metabolites. In the “Shanxi vs. others” group, Myristoylcarnitine and Sanggenone H were significantly upregulated. Ferulic acid was expressed at low levels in the Anhui group but at high levels in the Shanxi group. Notably, 6-Pentyl-2H-pyran-2-one entered the top 10 differential metabolites in both the Anhui and Shanxi groups, but the expression direction was completely opposite. In the “Henan vs. others” group, 7-Hydroxy-3-(2-hydroxy-propyl)-5-methyl-isochromen-1-one and cis-Aconitic acid were significantly downregulated, and the expression of Glechomafuran in the Henan group completely reversed the downward trend in the Anhui group. In all groups, 13-alpha-(21)-Epoxyeurycomanone showed highly significant differences, with its stable geographical response characteristics making it a potential key biomarker.

### 3.3. Machine Learning-Driven Screening System for Regional Biomarkers

**Figure 6:**
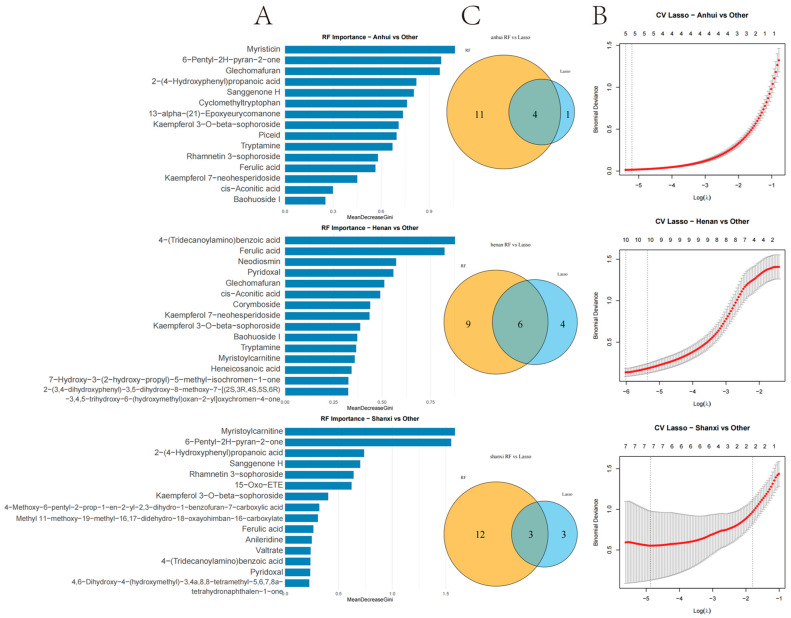
Machine learning-based screening of region-specific biomarkers in *T. chinense* Turcz. (**A**) RF importance ranking; (**B**) LASSO coefficient profiles for three regions; (**C**) Venn diagrams of RF-LASSO overlapping biomarkers.

By integrating Random Forest (RF) and Lasso regression algorithms (Figure 6), a dual screening system for the regional identification biomarkers of *T. chinense* Turcz. was established. In Anhui, the RF algorithm identified 15 compounds, while the Lasso algorithm identified five specific biomarkers, resulting in 4 overlapping compounds: Kaempferol 3-O-beta-sophoroside, 13-alpha-(21)-Epoxyeurycomanone, Myristicin, and Glechomafuran.

For Henan, the RF algorithm identified 15 marker compounds and the Lasso algorithm identified 10 characteristic components, resulting in six overlapping compounds: 7-Hydroxy-3-(2-hydroxy-propyl)-5-methyl-isochromen-1-one, Myristoylcarnitine, Kaempferol 3-O-beta-sophoroside, Kaempferol 7-neohesperidoside, Glechomafuran, and 4-(Tridecanoylamino)benzoic acid.

For Shanxi, the RF algorithm identified 15 biomarkers; the Lasso algorithm identified six specific compounds, resulting in three overlapping compounds: Rhamnetin 3-sophoroside, 6-Pentyl-2H-pyran-2-one, and Myristoylcarnitine. Kaempferol 3-O-beta-sophoroside, Glechomafuran, and Myristoylcarnitine were commonly identified in Anhui and Henan models, while Myristoylcarnitine was commonly identified in Shanxi and Henan models.

### 3.4. Pharmacological Mechanism Network of Geographically Specific Components

**Figure 7:**
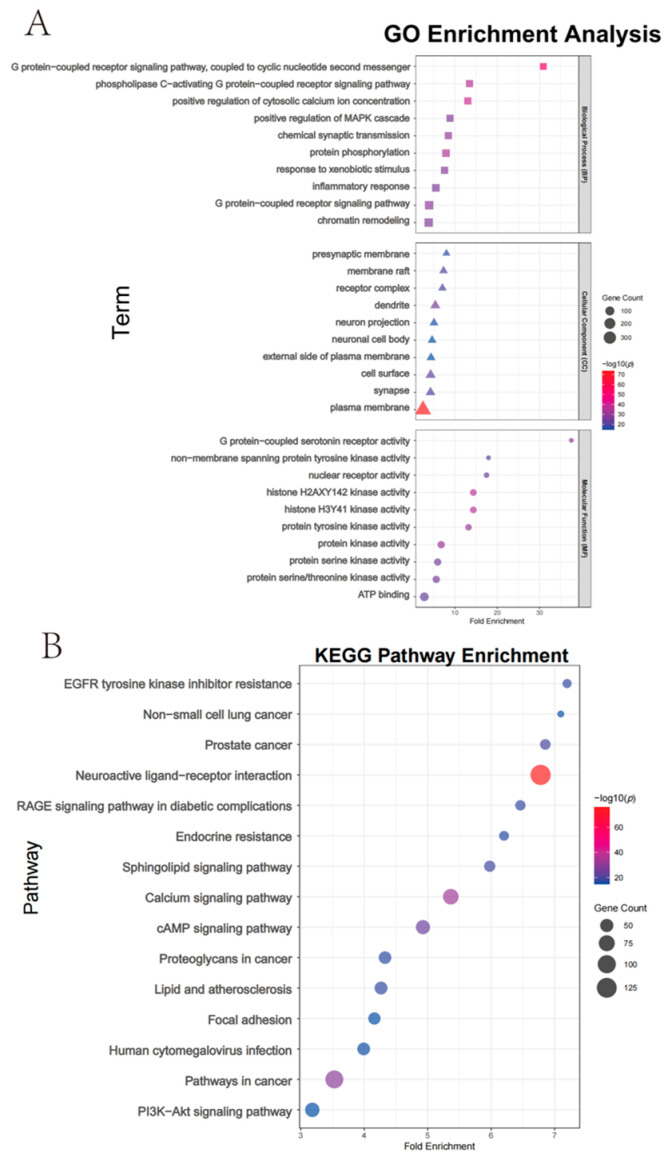
Functional enrichment analysis of *T. chinense* Turcz. biomarkers. (**A**) GO terms categorized into biological process (BP), cellular component (CC), and molecular function (MF); (**B**) KEGG pathway enrichment.

To elucidate the potential pharmacological mechanisms of region-specific biomarkers in *T. chinense* Turcz., key protein targets were predicted, and their biological functions were analyzed based on GO functional annotation and KEGG pathway enrichment analysis. GO analysis (Figure 7A) indicated that molecular function (MF) was mainly associated with histone H3Y41 kinase activity and G protein-coupled 5-hydroxytryptamine receptor activity, cellular component (CC) was concentrated in the plasma membrane and synapse, and biological process (BP) was dominated by G protein-coupled receptor signaling pathway and inflammatory response.

The 489 targets (Figure 7B) identified were significantly enriched in neuroactive ligand–receptor interaction, the calcium signaling pathway, and PI3K-Akt signaling pathway, with key receptor genes such as CHRM3, HTR2A, and DRD2 involved in the neuroactive pathway.

**Figure 8:**
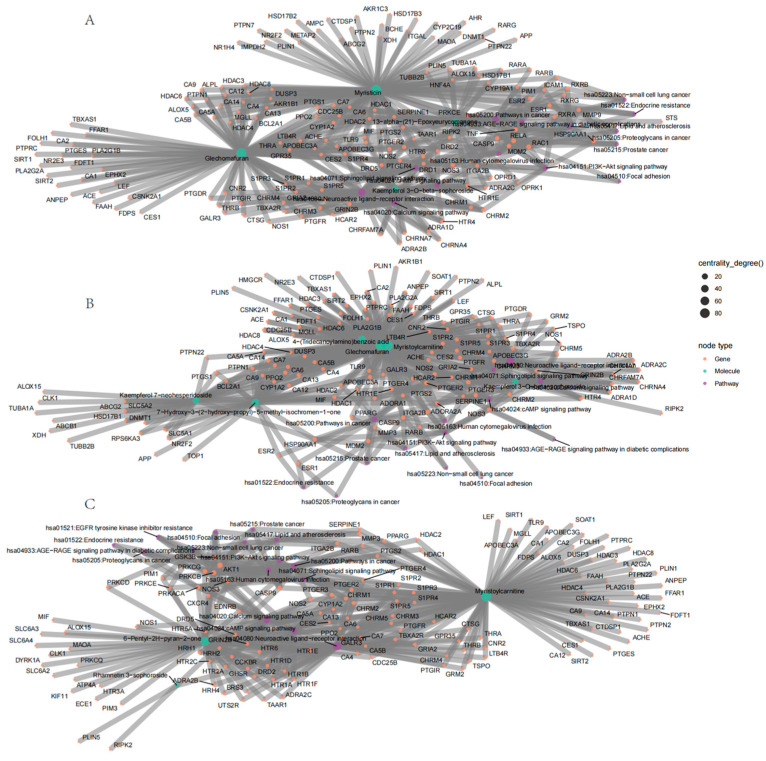
Region-specific compound–target–pathway networks: Anhui (**A**), Henan (**B**), and Shanxi (**C**).

The construction of region-specific “component–target–pathway” networks (Figure 8) revealed that in Anhui samples, Myristicin and Glechomafuran, significantly regulated the cholinergic receptor genes CHRM2/CHRM3/CHRM4, leading to enrichment in neuroactive ligand–receptor interaction pathways and calcium signaling pathways (Figure 8A). In Henan samples, Kaempferol 7-neohesperidoside and Myristoylcarnitine targeted ADORA1/PTGFR/PTGS2, showing a close association with lipid metabolism-related disease pathways (Figure 8B). Meanwhile, in Shanxi samples, Rhamnetin 3-sophoroside and 6-Pentyl-2H-pyran-2-one modulated kinase genes such as AKT1/PRKACA/GSK3B, specifically influencing EGFR tyrosine kinase inhibitor resistance pathways and the PI3K-Akt signaling pathway (Figure 8C). Notably, the gene NOS3 played a central role in pathway enrichment across all three regions (accounting for 62.3% of cross-regional targets), but its regulatory weight in the diabetes complication-related AGE-RAGE pathway exhibited significant regional differences (Anhui: 34 targets, Henan: 21 targets, Shanxi: 16 targets). Geographically specific compounds exert unique pharmacological effects by differentially regulating neurotransmitter receptors and metabolism-related pathways, with NOS3 potentially serving as a key regulatory hub for cross-regional synergistic effects.

### 3.5. Ecological Activity Correlation Model Construction and Validation

**Figure 9:**
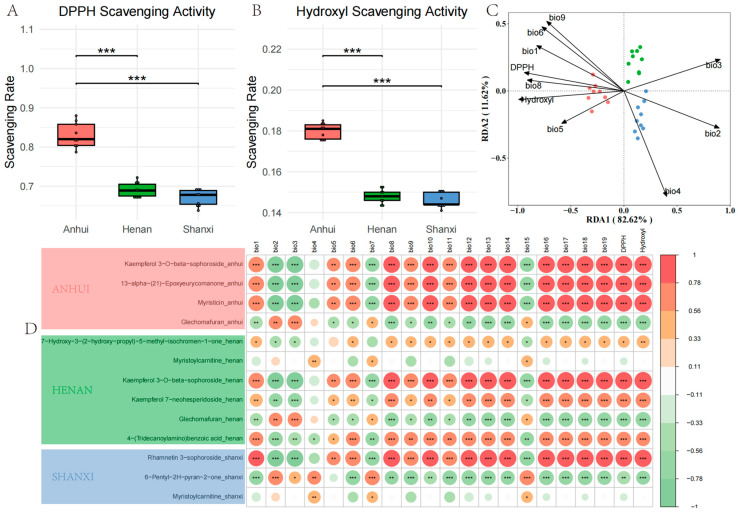
Antioxidant activity and eco-geographical correlation analysis of *T. chinense* Turcz. (**A**) DPPH radical scavenging rates; (**B**) hydroxyl radical inhibition rates; (**C**) RDA triplot of climatic factors, antioxidant activities, and biomarkers. The climatic variables used in the redundancy analysis (RDA) are based on the WorldClim Bioclimatic Variables and include the following: bio1: Annual mean temperature (°C), bio2: Mean diurnal range (mean of monthly (max temp—min temp)) (°C), bio3: Isothermality (bio2/bio7) (×100), bio4: Temperature seasonality (standard deviation × 100) (%), bio5: Max temperature of warmest month (°C), bio6: Min temperature of coldest month (°C), bio8: Mean temperature of wettest quarter (°C), bio9: Mean temperature of driest quarter (°C); (**D**) Pearson correlation heatmap of region-specific metabolites with geo-climatic variables and antioxidant indices. * *p* < 0.05, ** *p* < 0.01, *** *p* < 0.001, vs. Anhui group.

To reveal the geographical differentiation patterns of antioxidant activity in *T. chinense* Turcz. samples and the ecological regulation mechanisms of climate factors, antioxidant capacity was quantitatively assessed through DPPH and hydroxyl radical scavenging experiments. Anhui samples showed significantly higher DPPH scavenging rates (Figure 9A) and hydroxyl radical inhibition rates (Figure 9B) than Shanxi and Henan (*p* < 0.01), with Rhamnetin 3-sophoroside and 6-Pentyl-2H-pyran-2-one strongly positively correlated with antioxidant indices.

RDA (Figure 9C) indicated that temperature-related variables (bio1, bio8, bio9) and DPPH showed a significant negative correlation on RDA1, suggesting a close relationship between low-temperature environments and high DPPH activity. Precipitation indicators (bio4 temperature seasonality) dominated negatively on RDA2, suggesting that precipitation fluctuations may suppress antioxidant activity. The sample distribution showed Anhui samples concentrated on the negative axis of RDA1, consistent with their higher DPPH values, while Shanxi samples were dispersed along the negative axis of RDA2, corresponding to low hydroxyl activity.

Pearson correlation analysis (Figure 9D) revealed that Myristoylcarnitine in Shanxi was positively correlated with altitude (r = 0.63) and slope (r = 0.59), while Kaempferol 7-neohesperidoside in Henan was significantly negatively correlated with diurnal temperature range. The key component Kaempferol 3-O-beta-sophoroside in Anhui was extremely significantly positively correlated with DPPH activity, regulated positively by annual precipitation (bio12) and precipitation of the wettest month (bio13). Rhamnetin 3-sophoroside in Shanxi was significantly positively correlated with hydroxyl activity, indicating geographical adaptive metabolic characteristics. The correlation between 7-Hydroxy-3-(2-hydroxy-propyl)-5-methyl-isochromen-1-one in Henan and annual temperature range (bio7) was weak, suggesting a limited impact of annual temperature fluctuations on its synthesis.

The geographical differentiation of antioxidant activity is mainly regulated by temperature fluctuations and precipitation patterns, with mean annual temperature and seasonal precipitation being key factors driving the geographical differentiation of antioxidant activity in *T. chinense* Turcz. Low-temperature stable environments are conducive to enhancing DPPH activity, while drastic precipitation fluctuations in Shanxi may lead to reduced hydroxyl activity.

## 4. Discussion

This study systematically elucidates the geographical differentiation patterns of secondary metabolites in *T. chinense* Turcz. from Anhui, Henan, and Shanxi Provinces in China, as well as the underlying interaction mechanisms with ecological environmental factors, through the integration of multidimensional analytical methods. The metabolomic analysis based on LC-MS combined with machine learning algorithms not only confirms the decisive role of geographical environments in shaping the chemical characteristics of medicinal plants, as proposed by the traditional “geo-authentic herbs” theory, but also provides a quantifiable biomarker screening strategy for modern quality control systems of traditional Chinese medicine (TCM). The findings reveal the molecular mechanisms by which complex ecological factors regulate key metabolic pathways to influence plant chemical phenotypes, offering new theoretical insights into the environmental adaptability and evolutionary processes of medicinal plants.

The metabolomic analysis conducted in both positive and negative ion modes detected a total of 506 secondary metabolites, with 289 identified in positive ion mode and 217 in negative ion mode. PCA demonstrated a gradient distribution pattern of samples from the three production regions in the metabolic space. Samples from Henan and Shanxi partially overlapped along the PC1 and PC2 axes, indicating that shared ecological pressures or genetic homogeneity in these adjacent regions, consistent with reports on geographically proximate plant populations exhibiting convergent metabolic traits. From a climatic perspective, the sample collection sites in Anhui Province are situated south of the North–South climatic boundary, corresponding to a subtropical monsoon climate regime. In contrast, Henan and Shanxi Provinces are characterized by temperate monsoon climate conditions, characterized by comparable temperature ranges and precipitation patterns, which may homogenize flavonoid and alkaloid biosynthesis [21,22,23]. Notably, samples from Anhui exhibited distinct spatial clustering in positive ion mode, clearly distinguishing them from other regions. PLS-DA further validated these findings. This observation aligns with previous studies demonstrating that environmental factors such as altitude, temperature, and soil properties significantly influence secondary metabolite biosynthesis in medicinal plants.

The heatmap and enrichment analysis further identified 43 differential metabolites, dominated by flavonoids [19], alkaloids [12], and phenylpropanoids [8], and ten enriched pathways, with flavone/flavonol biosynthesis ranking highest. Flavonoids are well-documented for their antioxidant, anti-inflammatory, and neuroprotective properties [24,25,26], which correlate with the traditional use of *T. chinense* Turcz. in treating inflammatory disorders. The prominence of indole alkaloid pathways is equally significant, as these compounds exhibit antitumor and antimicrobial activities. Interestingly, the bidirectional regulation of kaempferol glycosides (e.g., kaempferol 7-neohesperidoside vs. kaempferol 3-O-beta-sophoroside) in Anhui samples implies tissue-specific enzymatic modifications, possibly influenced by regional soil micronutrients such as zinc or selenium, which modulate glycosyltransferase activity. This phenomenon underscores the plasticity of plant secondary metabolism in adapting to localized environmental cues, a phenomenon documented in other medicinal plants [27] such as Ginkgo biloba.

The comparative PLS-DA models (Anhui vs. others, Shanxi vs. others, Henan vs. others) and metabolite set enrichment analysis identified distinct regional metabolic signatures, with Anhui samples exhibiting the most unique chemical characteristics. Myristicin—a phenylpropene with known acetylcholinesterase inhibitory effects [28]—was enriched in Anhui but downregulated in Shanxi. This contrast may reflect differences in soil nitrogen availability, as phenylpropanoid biosynthesis is nitrogen-dependent [29]. Similarly, the reversal of ferulic acid trends between Anhui (downregulated) and Shanxi (upregulated) could be attributed to ultraviolet (UV) radiation intensity, which stimulates ferulic acid synthesis as a photoprotective mechanism [30]. Shanxi’s higher altitude and stronger UV exposure align with this hypothesis. Glechomafuran and Cyclomethyltryptophan were downregulated in Anhui samples but upregulated in Shanxi populations. This cross-regional expression divergence likely reflects adaptive responses to local climatic conditions, particularly temperature fluctuations and precipitation patterns. Similar geographical regulation of secondary metabolites has been observed in Saussurea involucrata leaves, where low-temperature environments enhance phenolic compound accumulation [31]. The reverse regulation of 6-Pentyl-2H-pyran-2-one in the Anhui and Shanxi samples highlights the complexity of ecological adaptation. This compound, known for its antimicrobial properties [32], may serve as a stress-response metabolite under contrasting environmental pressures. The isomer-specific regulation of kaempferol glycosides further highlights the role of environmental factors in steering metabolic branching. The 82% decrease in kaempferol 7-neohesperidoside and 3.2-fold increase in its isomer in Anhui samples suggest that temperature fluctuations may influence glycosyltransferase specificity. Additionally, the consistent significance of 13-alpha-(21)-Epoxyeurycomanone across all regions positions it as a universal biomarker for *T. chinense* Turcz.

The integration of Random Forest and Lasso regression algorithms identified overlapping biomarkers with high diagnostic value for geographical discrimination. This dual-algorithm approach outperforms traditional univariate analyses by capturing nonlinear relationships and reducing overfitting risks. Among the Anhui markers, Kaempferol 3-O-beta-sophoroside, 13-alpha-(21)-Epoxyeurycomanone, Myristicin, and Glechomafuran exhibited significant regional specificity. Myristicin and Glechomafuran were significantly enriched in neuroactive ligand–receptor interaction and calcium signaling pathways, suggesting their roles in modulating neurotransmitter regulation and calcium homeostasis through genes like CHRM2/CHRM3/CHRM4. Among the six intersection compounds in Henan, the specific accumulation of 7-Hydroxy-3-(2-hydroxy-propyl)-5-methyl-isochromen-1-one and 4-(Tridecanoylamino)benzoic acid may reflect the diversification of secondary metabolic pathways due to complex terrain. Kaempferol 7-neohesperidoside and Myristoylcarnitine were primarily enriched in lipid metabolism-related pathways, implicating their roles in regulating lipid metabolism via targets such as ADORA1/PTGFR/PTGS2. This aligns with the lower annual precipitation in Henan, as drought stress often elevates lipid-derived metabolites to maintain membrane integrity. Among the three intersection compounds in Shanxi, the significant upregulation of Rhamnetin 3-sophoroside and 6-Pentyl-2H-pyran-2-one affected EGFR tyrosine kinase inhibitor resistance and PI3K-Akt signaling pathways by modulating kinase genes like AKT1/PRKACA/GSK3B, likely driven by high-altitude oxidative stress. Cross-regional comparisons revealed that Kaempferol 3-O-beta-sophoroside, Glechomafuran, and Myristoylcarnitine were consistently identified across multiple models, demonstrating robust regional differentiation capabilities. In the multi-regional “component–target–pathway” integrated network, the NOS3 gene played a central role in pathway enrichment across all three regions, accounting for 62.3% of cross-regional targets. However, its regulatory weight in the AGE-RAGE pathway associated with diabetic complications varied significantly across regions: 34 targets in Anhui, 21 in Henan, and 16 in Shanxi. This variation suggests differences in the functional mechanisms of NOS3 among regions, but its status as a potential key regulatory hub for cross-regional synergy remains noteworthy.

RDA and Pearson correlation models elucidated the climatic and topographical drivers of antioxidant activity. Anhui’s superior DPPH and hydroxyl radical scavenging rates were tightly linked to Rhamnetin 3-sophoroside and 6-Pentyl-2H-pyran-2-one, which correlated positively with annual precipitation (bio12) and negatively with temperature seasonality (bio4). This mirrors findings in Camellia sinensis, where stable rainfall enhances phenolic accumulation [33]. Conversely, Shanxi’s reduced hydroxyl activity under precipitation fluctuations (bio4) may reflect trade-offs between antioxidant synthesis and drought resilience mechanisms. The inverse correlation between temperature variables (bio1, bio8) and DPPH activity underscores the role of cool climates in promoting flavonoid biosynthesis, as low temperatures upregulate phenylalanine ammonia-lyase (PAL) activity [34]. Similarly, the altitude-dependent enrichment of Myristoylcarnitine in Shanxi (r = 0.63) suggests adaptive lipid metabolism to counter hypoxia-induced oxidative stress, a phenomenon observed in high-altitude medicinal plants. The negative correlation between Kaempferol 7-neohesperidoside in Henan samples and diurnal temperature variation suggests the regulatory effect of temperature stability on flavonoid glycosyltransferase activity.

The quantitative integration of climate variables with specific metabolites provides predictive models for cultivation optimization, enhanced by the RF-LASSO pipeline’s superior specificity in reducing false-positive biomarker identification. However, limitations include restricted sampling across three provinces and single-year data collection, necessitating broader geographic and longitudinal studies to assess biomarker universality and climate change impacts on metabolite stability, alongside the incorporation of rhizosphere microbiome analyses through metagenomics to elucidate plant-microbe co-metabolism effects. For quality control and cultivation optimization, actionable strategies emerge: Anhui Province should prioritize low-temperature/high-precipitation microclimates to maximize neuroactive flavonoids (e.g., Kaempferol glycosides), Shanxi Province can enhance alkamide production (Myristoylcarnitine) by targeting high-soil organic carbon zones, and Henan Province requires diurnal temperature management to balance lipid-regulating and antioxidant metabolites, with stable biomarkers like 13-alpha-Epoxyeurycomanone serving as key quality indicators.

## 5. Conclusions

This study systematically investigates the geographical chemodiversity of *T. chinense* Turcz. and its underlying ecological–molecular mechanisms. LC-MS metabolomics revealed significant regional differentiation in secondary metabolite profiles, with samples from Anhui forming a distinct cluster in positive ion mode. Comparative analysis identified 43 geographically differential metabolites, primarily flavonoids, alkaloids, and phenylpropanoids. Functional analysis indicated that these differential metabolites target critical pathways, including neuroactive ligand–receptor interactions, lipid metabolism, and EGFR tyrosine kinase inhibitor resistance. NOS3 emerged as a cross-regional hub gene modulating diabetes-related AGE-RAGE pathways, exhibiting demonstrable regional specificity. RDA and Pearson correlation confirmed strong associations between metabolomic profiles and regional climatic/edaphic factors. Enhanced stability at lower temperatures correlated with increased DPPH radical scavenging capacity in Anhui samples, while precipitation seasonality significantly elevated hydroxyl radical scavenging activity in Shanxi samples.

Collectively, these findings validate the scientific basis of the traditional “geo-authentic” medicinal material theory. It provides theoretical support for molecular breeding programs, enhanced quality control standards, and the sustainable exploitation of medicinal plants. Future studies should employ multi-omics approaches to further decipher the environmental stress response mechanisms governing these metabolic pathways.

## Figures and Tables

**Table 1:** Environmental variables recorded for each sample area.

Field	Description	Units
bio1	annual mean temperature	°C
bio2	mean diurnal range (mean of monthly (max temp—min temp))	°C
bio3	isothermality (bio2/bio7) (×100)	°C
bio4	temperature seasonality (standard deviation × 100)	%
bio5	max temperature of warmest month	°C
bio6	min temperature of coldest month	°C
bio7	temperature annual range(bio5-bio6)	°C
bio8	mean temperature of wettest quarter	°C
bio9	mean temperature of driest quarter	°C
bio10	mean temperature of warmest quarter	°C
bio11	mean temperature of coldest quarter	°C
bio12	annual precipitation	mm
bio13	precipitation of wettest month	mm
bio14	precipitation of driest month	mm
bio15	precipitation seasonality (coefficient of variation)	%
bio16	precipitation of wettest quarter	mm
bio17	precipitation of driest quarter	mm
bio18	precipitation of warmest quarter	mm
bio19	precipitation of coldest quarter	mm

## Data Availability

The original contributions presented in this study are included in the article; further inquiries can be directed to the corresponding author.

## Abbreviations

The following abbreviations are used in this manuscript:

TCM	Traditional Chinese Medicine
LC-MS	Liquid Chromatography-Mass Spectrometry
UPLC-QTOF-MS	Ultra-Performance Liquid Chromatography-Quadrupole Time-of-Flight Mass Spectrometry
PCA	Principal Component Analysis
PLS-DA	Partial Least Squares Discriminant Analysis
VIP	Variable Importance in Projection
RF	Random Forest
LASSO	Least Absolute Shrinkage and Selection Operator
RDA	Redundancy Analysis
DPPH	2
HMDB	Human Metabolome Database
KEGG	Kyoto Encyclopedia of Genes and Genomes
GO	Gene Ontology
ANOVA	Analysis of Variance
ROS	Reactive Oxygen Species
NOS3	Nitric Oxide Synthase 3
AGE-RAGE	Advanced Glycation End Products-Receptor for Advanced Glycation End Products
EGFR	Epidermal Growth Factor Receptor
PI3K-Akt	Phosphatidylinositol 3-Kinase-Protein Kinase B
ADORA1	Adenosine A1 Receptor
PTGFR	Prostaglandin F2α Receptor
PTGS2	Prostaglandin-Endoperoxide Synthase 2
AKT1	Protein Kinase B Alpha
PRKACA	Protein Kinase A Catalytic Subunit Alpha
GSK3B	Glycogen Synthase Kinase 3 Beta
CHRM2/CHRM3/CHRM4	Cholinergic Receptor Muscarinic 2/3/4
HTR2A	5-Hydroxytryptamine Receptor 2A
DRD2	Dopamine Receptor D2
CHRM3	Cholinergic Receptor Muscarinic 3
